# A national-scale vegetation multimetric index (VMMI) as an indicator of wetland condition across the conterminous United States

**DOI:** 10.1007/s10661-019-7324-4

**Published:** 2019-06-20

**Authors:** Teresa K. Magee, Karen A. Blocksom, M. Siobhan Fennessy

**Affiliations:** 1 Office Research and Development, National Health and Environmental Effects Research Laboratory, Western Ecology Division, U.S. Environmental Protection Agency, 200 SW 35th Street, Corvallis, 97333 OR USA; 2 Biology Department, Kenyon College, Gambier, OH USA

**Keywords:** Vegetation multimetric index (VMMI), Ecological condition, National Wetland Condition Assessment, Floristic quality, Wetland monitoring, Permutation methods

## Abstract

In 2011, the US Environmental Protection Agency and its partners conducted the first National Wetland Condition Assessment at the continental-scale of the conterminous United States. A probability design for site selection was used to allow an unbiased assessment of wetland condition. We developed a vegetation multimetric index (VMMI) as a parsimonious biological indicator of ecological condition applicable to diverse wetland types at national and regional scales. Vegetation data (species presence and cover) were collected from 1138 sites that represented seven broad estuarine intertidal and inland wetland types. Using field collected data and plant species trait information, we developed 405 candidate metrics with potential for distinguishing least disturbed (reference) from most disturbed sites. Thirty-five of the metrics passed range, repeatability, and responsiveness screens and were considered as potential component metrics for the VMMI. A permutation approach was used to calculate thousands of randomly constructed potential national-scale VMMIs with 4, 6, 8, or 10 metrics. The best performing VMMI was identified based on limited redundancy among constituent metrics, sensitivity, repeatability, and precision. This final VMMI had four broadly applicable metrics (floristic quality index, relative importance of native species, richness of disturbance-tolerant species, and relative cover of native monocots). VMMI values and weights from the survey design for probability sites (*n* = 967) were used to estimate wetland area in good, fair, and poor condition, nationally and for each of 10 ecoregion by wetland type reporting groups. Strengths and limitations of the national VMMI for describing ecological condition are highlighted.

**Electronic supplementary material:**

The online version of this article (10.1007/s10661-019-7324-4) contains supplementary material, which is available to authorized users.

## Introduction

The National Wetland Condition Assessment (NWCA) (USEPA 2016m) provided an unprecedented opportunity to characterize the quality of wetlands at a continental-scale. The 2011 NWCA was conducted by the US Environmental Protection Agency (USEPA) and its partners (states, tribes, other federal agencies, universities, and other organizations), and represents the first assessment of wetland condition that spans the conterminous United States (US). Primary goals of the NWCA were to evaluate the ecological condition of wetlands in the US (this paper; Kentula and Paulsen 2019) and identify stressors that might affect condition (Herlihy et al. 2019b, c; Lomnicky et al. 2019; Magee et al. 2019; Nahlik et al. 2019). The survey was designed to assess the condition of broad groups or subpopulations of wetlands at national or regional scales, rather than the condition of individual wetlands or wetlands across individual states (Olsen et al. 2019). The NWCA is the fourth in a series of National Aquatic Resource Surveys (NARS), with previous assessments focusing on rivers and streams, lakes and reservoirs, and near coastal areas (e.g., USEPA 2006, 2009, 2015, 2016l).

All NARS employ a probability design for site selection, which permits inference to national and regional scales (Stevens and Olsen 2004; Olsen and Peck 2008; Olsen et al. 2019). Ecological condition is estimated using biological indicators, typically multimetric indices (MMI) based on the combination of a few easily measured and interpreted metrics that describe different attributes of a particular biotic assemblage (e.g., USEPA 2006; Stoddard et al. 2008; USEPA 2009). These indices vary with human-mediated disturbance (hereafter, disturbance), reflecting departure from reference expectations that are typically defined based on the least disturbed sites within a study area (Stoddard et al. 2006; Herlihy et al. 2008). Indices of Biotic Integrity (IBIs) are similar to MMIs, but are often constructed using biology-driven expert judgment for metric selection (e.g., Karr 1991), whereas MMIs are based on a more data-driven approach for selecting component metrics (e.g., Stoddard et al. 2008). However, for wetlands, the term IBI has often been applied to both the traditional IBI and the more objective MMI approaches.

MMIs and IBIs have been widely used as cost-effective indicators of biological condition in various aquatic ecosystems (Karr 1991; Karr and Chu 1997; Jackson et al. 2000; Dale and Beyeler 2001; Stoddard et al. 2008) and have been developed for diverse biotic assemblages (e.g., fish and amphibians (Hughes et al. 2004; Whittier et al. 2007; Micacchion et al. 2015), aquatic macroinvertebrates (Herlihy et al. 2005), algae (Fetscher et al. 2014), birds (Bryce and Hughes 2002; Bryce 2006), mosses (Stapanian et al. 2016), riparian vegetation (Ferreira et al. 2005; Aguiar et al. 2009), and wetland vegetation (Mack and Kentula 2010)). Neither MMIs nor IBIs are intended as comprehensive descriptors of all components of ecological condition, or even all aspects of the particular biotic assemblage on which they are based, but they have proven to illustrate clear and reliable trends in condition across diverse sample populations.

For the 2011 NWCA, our goal was to develop a MMI based on vascular vegetation (VMMI) as a biological indicator of wetland condition. Vascular plant species represent diverse adaptations, ecological tolerances, and life history strategies, and they integrate environmental factors, species interactions, and disturbance. Many disturbances are reflected in shifts in the presence or abundance of particular plant species (Magee and Kentula 2005; Johnston et al. 2008), plant functional or trait groups (Lopez and Fennessy 2002; Quétier et al. 2007), plant assemblages (Galatowitsch et al. 1999; Magee et al. 1999; DeKeyser et al. 2009; Johnston et al. 2009), or vegetation structural elements (Mack 2007), making vegetation a powerful indicator of wetland condition (Mack and Kentula 2010). Existing VMMIs or VIBIs have proven useful for monitoring condition and prioritizing conservation or management actions for specific wetland types at local or regional scales within the United States and elsewhere (e.g., DeKeyser et al. 2003; Miller et al. 2006; Reiss 2006; Mack 2007; Hargiss et al. 2008; Rothrock et al. 2008; Lemly and Rocchio 2009; Mack 2009; Veselka et al. 2010; Euliss and Mushet 2011; Genet 2012; Rooney and Bayley 2012; Deimeke et al. 2013; Wilson et al. 2013; Hernandez et al. 2015; Savage et al. 2015; Jones et al. 2016; Miller et al. 2016).

Our challenge was to produce one national-scale VMMI with applicability to diverse wetland types across the conterminous US that could provide a consistent approach to evaluating wetland quality across very large scales. Ideally, a national-scale VMMI should respond negatively to disturbance and be robust enough to represent condition in different wetland types and regions across the continent. Criteria for developing and choosing an effective VMMI for the NWCA were that it should (1) reflect condition relative to vegetation at least disturbed sites; (2) be parsimonious, i.e., based on the smallest number of easy-to-measure vegetation metrics that satisfactorily represent condition; and (3) account for biotic variability related to natural environmental gradients or to regional differences in least disturbed condition. A common procedure for constructing VMMIs parallels approaches for MMI development used by NARS (Stoddard et al. 2008). First, many individual candidate metrics of vegetation condition are screened based on several criteria (e.g., range, repeatability, responsiveness, and redundancy). Next, a suite of the most effective metrics is combined into a single VMMI scaled on a continuous range of 0 to 100. Finally, thresholds for observed VMMI values are set to connote good, fair, and poor wetland condition based on the distribution of VMMI values among independently identified least disturbed sites. We used an adaptation of this general approach (sensu Van Sickle 2010) in developing the NWCA VMMI.

In this paper, we describe our methods for (1) developing a national-scale VMMI and (2) setting VMMI thresholds for categories of condition (good, fair, and poor) for 10 ecoregion by wetland type reporting groups that together encompass the continental US. Thresholds for condition categories are set within reporting groups to represent natural differences in the VMMI that may be related to attributes of ecoregions or wetland types. We apply the final wetland VMMI to the vegetation data collected in the 2011 NWCA to obtain baseline estimates of wetland area currently in good, fair, and poor conditions across the conterminous US and for subpopulations based on the 10 reporting groups. Finally, we discuss some of the strengths and limitations of the VMMI as an indicator of wetland condition and next steps for moving forward in the next iterations of the NWCA.

## Methods

### NWCA target population and survey design

Wetlands were defined, for the NWCA, as lands transitional between terrestrial and aquatic systems where the water table is frequently at or near the surface or the land is covered by shallow water (Cowardin et al. 1979; USEPA 2016m). The jurisdictional status of wetlands under state or federal regulatory programs was not considered in this definition. The NWCA target wetland population included seven broad wetland types of the conterminous US (Table 1), which encompassed tidal and nontidal systems with rooted vegetation and, when present, open water less than 1 m deep (USEPA 2011a, 2016m).

**Table 1:** Definition of NWCA target population and the seven included NWCA Wetland Types, and description of the aggregation of these types for analysis

Target Population		NWCA wetland type	Aggregated type	Description
Wetlands across conterminous United States representing tidal and nontidal systems with rooted vegetation and, *when present*, *open water ≤ 1-m deep*	Estuarine intertidal	EH—Estuarine intertidal emergent EW—Estuarine intertidal shrub/forest	EH—Estuarine herbaceous EW—Estuarine woody	Estuarine or intertidal emergent wetlands Estuarine or intertidal shrub and forested wetlands
	Inland	PRL-EM—Palustrine, riverine, or lacustrine emergent PRL-UBAB—Palustrine, riverine, or lacustrine unconsolidated bottom/aquatic bed PRL-f—Palustrine, riverine, or lacustrine farmed (not actively farmed)	PRLH—Palustrine, riverine, and lacustrine herbaceous	Emergent, ponded, or previously farmed wetlands in palustrine, shallow riverine, or shallow lacustrine littoral settings
		PRL-SS—Palustrine, riverine, or lacustrine shrub/scrub PRL-FO—Palustrine, riverine, or lacustrine forested	PRLW—Palustrine, riverine, and lacustrine woody	Forest or shrub dominated wetlands in palustrine, shallow riverine, or shallow lacustrine littoral settings

The NWCA survey design is detailed in Olsen et al. (2019) and summarized here. Sites were selected from the US Fish & Wildlife Service’s (USFWS) National Wetland Status and Trends (S&T) digital sample frame for wetlands (Dahl and Bergeson 2009; Dahl 2011) using a spatially balanced, unequal probability, Generalized Random Tessellation Stratified (GRTS) design for an area resource (Stevens and Olsen 2004, Olsen et al. 2012). The NWCA target population represented a major subset of the wetland categories included in the S&T sample frame, but excluded S&T categories that typically lack vegetation or routinely occur in deep water (e.g., Marine Intertidal or Subtidal, Estuarine Intertidal Unconsolidated Shore or Aquatic Bed) (USEPA 2016m, 2016n). Key elements of the survey design were (1) stratification by state with sites proportionally allocated by area of NWCA wetland types, (2) a minimum of eight sites per state, and (3) sufficient sample size for reporting on wetland condition nationally and within various subpopulations. Each selected sample point (i.e., coordinates of site location) received a weight that reflected the acres of wetland in the target population represented by that point (Olsen et al. 2019). Sample-weights were used to estimate wetland area (Diaz-Ramos et al. 1996) across the nation, regionally, or by wetland type with a known margin of error based on a local neighborhood variance estimate (Stevens and Olsen 2003). Site selection, weight assignment, and wetland area estimation were completed using the R statistical software (R Core Team 2015) and the “spsurvey” R contributed package (Kincaid and Olsen 2016).

Results from the survey design estimated the area of the NWCA target population across the conterminous US, in 2011, at 38.4 ± 2.51 million hectares (Olsen et al. 2019). Approximately 1/3 of this area was represented by sites that could not be sampled due to denial of access by land owners (*n* = 429), inaccessibility (*n* = 126; safety concerns or remote location), or other constraints (*n* = 122; e.g., too near another sampling point, crossing hydrogeomorphic boundaries, sampling area too small). Unassessed sites cannot be assumed to be randomly distributed; consequently, results of the 2011 NWCA could not be extrapolated to this portion of target population (USEPA 2016n). During the 2011 field season, 967 probability sites were sampled, and these 967 sites represent the inference or sampled population to which results of this study are applicable, an estimated 25.15 ± 2.27 million hectares of wetlands (Olsen et al. 2019).

In addition to the 967 probability sites from the NWCA design, another 171 sites were selected using non-NWCA design approaches. In an effort to supplement the number of least disturbed sites that might be observed in the probability sample, 150 handpicked sites that passed a series of landscape-level or best professional judgment screens were sampled to try to identify potentially high quality reference sites (but, see Herlihy et al. 2019a). Finally, 21 other sites were sampled as part of the NWCA for various state-level studies (USEPA 2016n). All 1138 sampled sites were evaluated using quantitative screening criteria to determine disturbance status as least (reference), intermediate, or most disturbed (see section “Reference expectations and disturbance categories”).

**Fig. 1:**
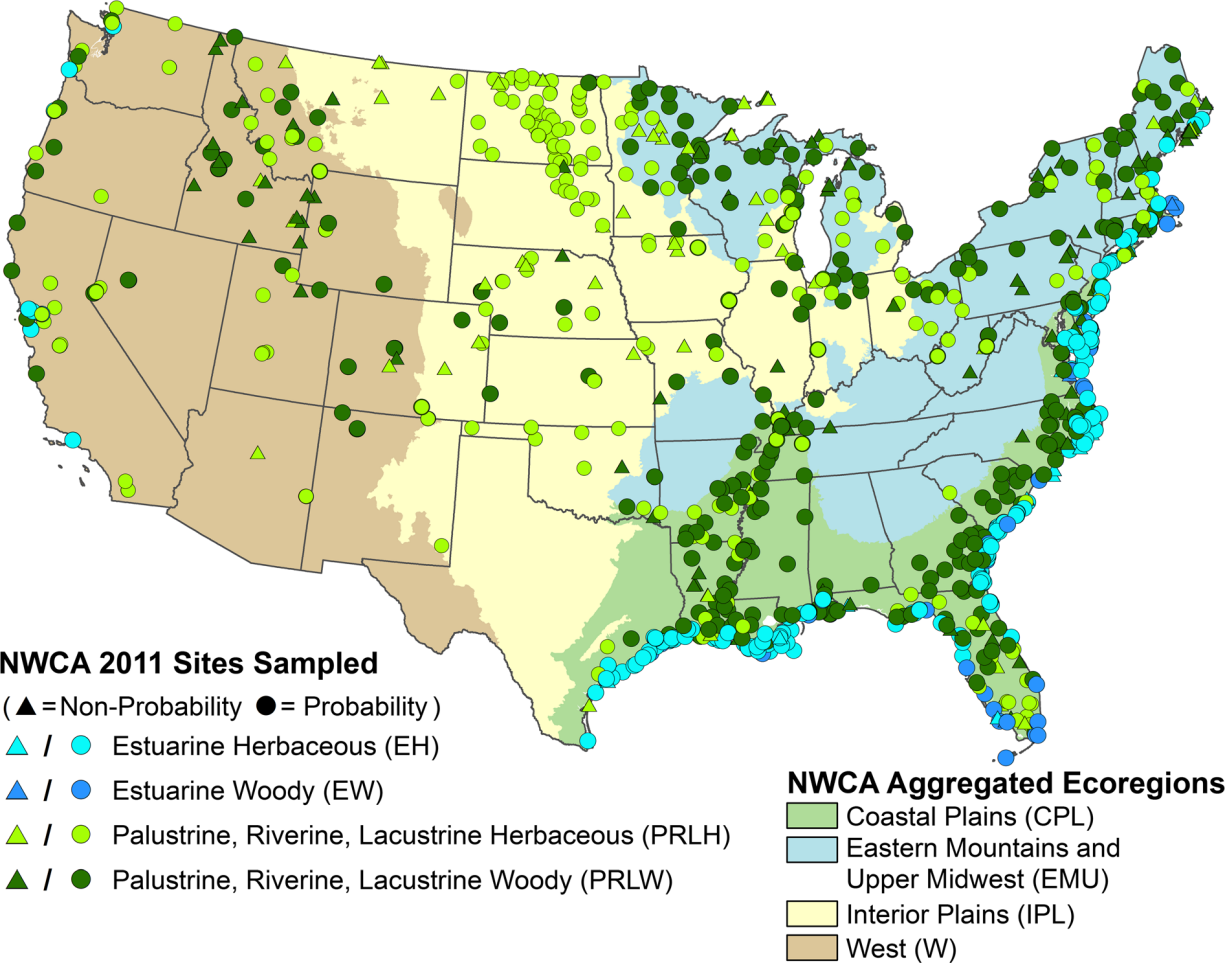
Study area map. Locations of sites sampled in the 2011 National Wetland Condition Assessment (NWCA) by aggregated wetland type (see Table 2) within the four NWCA Ecoregions (USEPA 2016n). Note, due to map scale and site proximity, individual sites are occasionally obscured by symbols for other sites

In this study, all 1138 sampled sites (Fig. 1, see also Online Resource 1—gray scale version of map) were used in VMMI development; however, estimates of wetland area with particular characteristics (e.g., condition category, ecoregion, wetland type) were based only on the 967 probability sites. Among probability sites, 96 were identified as part of the survey design for repeat sampling during the field season (revisit sites) to allow assessment of within-year sampling variability. A subset of NWCA site information data (USEPA 2016f; site identifiers, location, sample-weights, and status within various classifications (e.g., ecoregions, wetland types)) was used to support analyses presented in this paper.

### Natural variation and reporting groups

At the scale of the conterminous United States, natural variation in plant species composition and environmental conditions related to ecoregion and wetland type has implications for identifying reference sites and setting expectations for biotic condition. In large-scale condition assessments, ecoregional or typological classifications or modeling approaches have often been employed to adjust for this variation (e.g., Stoddard 2004; Stoddard et al. 2006, 2008; Herlihy et al. 2008; Pont et al. 2009; Hawkins et al. 2010; Minnesota Pollution Control Agency 2015; Jones et al. 2016). For the NWCA, a classification hierarchy was developed to (1) help account for continental-scale differences in wetland vegetation and environmental factors and (2) define groups that maximize within-class similarity and maintain sufficient sample size for characterizing reference expectations and evaluating ecological condition (Herlihy et al. 2019a). This classification was informed by vegetation patterns determined by species composition, and defines 10 ecoregion *×* wetland type groups (hereafter reporting groups (Table 2)) based on combinations of the four aggregated ecoregions (Fig. 1) and four aggregated wetland types (encompassing the seven NWCA wetland types of the target population, Table 1). The aggregated wetland types include estuarine herbaceous (EH) and woody (EW) wetlands, and inland (palustrine, shallow riverine, or shallow lacustrine) herbaceous (PRLH) and woody (PRLH) wetlands. Separate reporting groups (*n* = 8) were defined for herbaceous and woody inland types by NWCA ecoregion (Table 2). Estuarine intertidal systems occurred in seaward coastal areas of the US (Fig. 1), but were much more common in the Coastal Plains (CPL, *n* = 306) ecoregion than in the Eastern Mountains and Upper Midwest (EMU, *n* = 14) or the West (W, *n* = 25) ecoregions. Due to sample size limitations in the EMU and W, estuarine wetlands were grouped across the CPL, EMU, and W ecoregions and classed into two reporting groups based on aggregated wetland type: all-estuarine intertidal herbaceous (ALL-EH) and all-estuarine intertidal woody (ALL-EW) (Table 2). The 10 NWCA reporting groups were used for defining reference expectations, informing development of the VMMI, and reporting results.

**Table 2:** Distribution of the 1138 sites (967 probability + 171 non-probability) sampled in the 2011 NWCA nationally, for calibration and validation data, and by reporting group, including total number of sites, number of sites within disturbance category, and number of revisit sites

NWCA data subset		Total	Least disturbed	Intermediate disturbance	Most disturbed	Revisit^b^
Nationally	All sites^a^	1138	277	529	332	96
	Calibration data	911	222	423	266	78
	Validation data	227	55	106	66	18
Reporting groups (ecoregion^c^ × wetland type^d^)						
ALL-EH	All—Estuarine intertidal herbaceous	272	100	90	82	18
ALL-EW	All—Estuarine intertidal woody	73	16	38	19	3
CPL-PRLH	Coastal Plain—Palustrine, riverine, or lacustrine herbaceous	72	16	36	20	3
CPL-PRLW	Coastal Plain—Palustrine, riverine, or lacustrine woody	189	37	97	55	11
EMU-PRLH	Eastern Mountains and Upper Midwest—Palustrine, riverine, or lacustrine herbaceous	73	16	33	24	10
EMU-PRLW	Eastern Mountains and Upper Midwest—Palustrine, riverine, or lacustrine woody	127	21	79	27	15
IPL-PRLH	Interior Plains—Palustrine, riverine, or lacustrine herbaceous	138	26	70	42	16
IPL-PRLW	Interior Plains—Palustrine, riverine, or lacustrine woody	52	12	26	14	3
W-PRLH	West—Palustrine, riverine, or lacustrine herbaceous	75	17	30	28	9
W-PRLW	West—Palustrine, riverine, or lacustrine woody	67	16	30	21	8

^a^Rows in table represent subsets of the all sites totals

^b^All revisit sites were sampled twice and were probability sites

^c^See Fig. 1 for NWCA ecoregion boundaries

^d^See Table 1 for definition of aggregated wetland types

### Reference expectations and disturbance categories

A set of reference sites and a set of most disturbed sites are needed for MMI development (Stoddard et al. 2008). Unfortunately, local- and global-scale human-caused changes to the environment have resulted in pristine natural conditions being rare or absent in most locations, so it is often necessary to characterize reference condition in relation to the least disturbed sites in a sampled population (Stoddard et al. 2006; Herlihy et al. 2008, 2013). This approach for defining reference expectations for potential VMMIs was adopted for the NWCA and we use the terms “reference” and “least disturbed” interchangeably throughout this paper. The most disturbed sites were identified relative to the highest disturbance level observed in a particular sampled population.

**Fig. 2:**
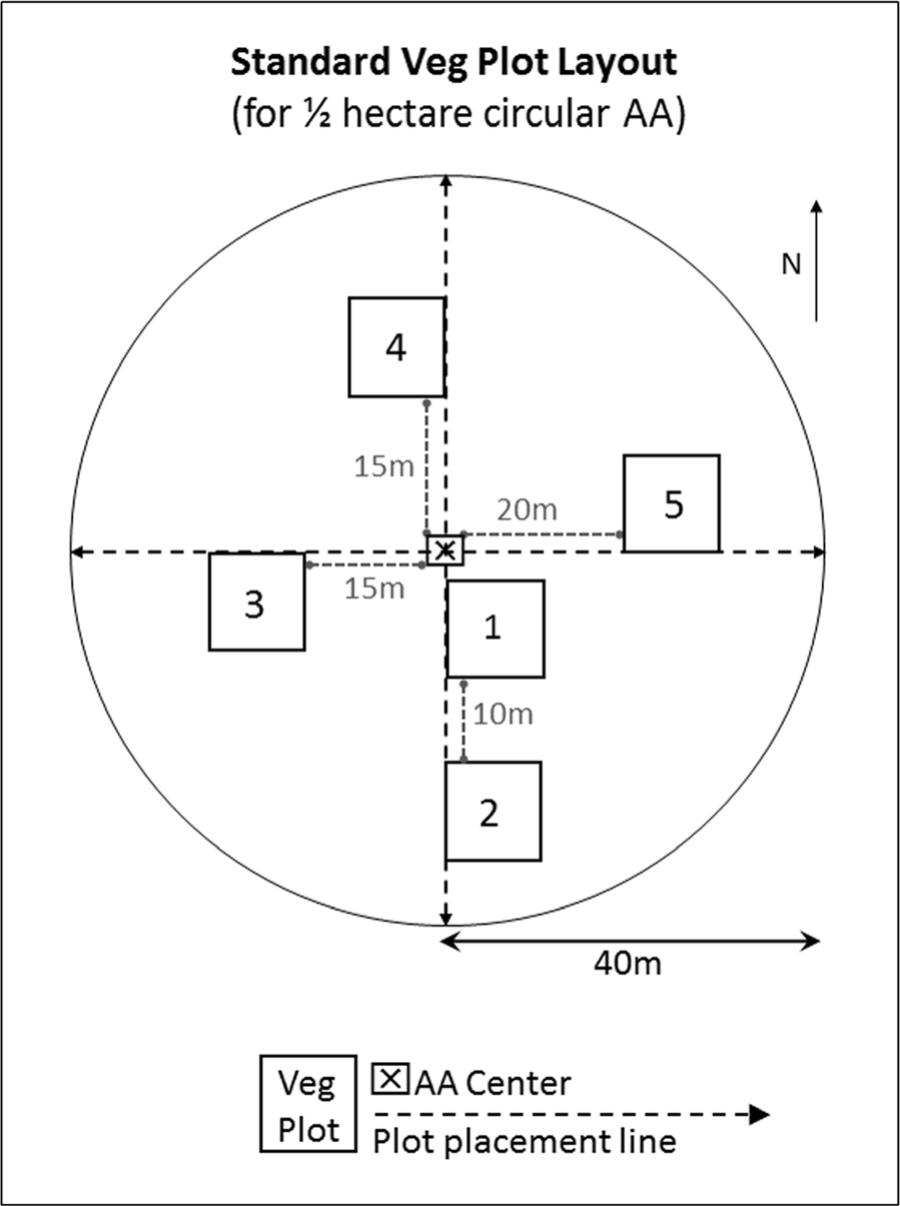
Vegetation plot layout for standard circular assessment area (AA). All features drawn to scale

Both least and most disturbed sites for the NWCA were identified by Herlihy et al. (2019a) using specific quantitative criteria, which are briefly summarized here. The presence, abundance, or severity of 85 specific descriptors of disturbance were characterized based on data or samples collected at each of the 1138 sampled sites, within the Assessment Area (AA) (Fig. 2) and/or a spatial buffer (typically a radius of 100 m) surrounding the AA (USEPA 2011a, 2016n). Disturbance was quantified using ten site-level indices derived from these field descriptors (see Herlihy et al. 2019a and disturbance index data: USEPA 2016a). Six of the indices summarized categories of physical disturbances (agriculture, residential/urban, industrial, hydrologic modifications, habitat modifications, overall disturbance) in the AA and buffer area and two described the impact level of hydrologic alterations in the AA (Lomnicky et al. 2019). One disturbance index described heavy metals in the soil in the AA (Nahlik et al. 2019) and one quantified relative cover of alien plant species in the AA (USEPA 2016n).

The level of disturbance observed for the NWCA sampled sites, based on these ten indices, varied by reporting group; thus, separate reporting group-specific screening thresholds for least and most disturbed status were developed for each disturbance index (Herlihy et al. 2019a). Herlihy et al. (2019a) assigned each sampled site to a disturbance category, such that (1) reference sites were identified as those where all ten disturbance index values fell below reporting group thresholds for least disturbed status, whereas exceeding the least disturbed threshold for any index disqualified a site from least disturbed status; (2) exceeding the most disturbed threshold for any one of the disturbance metrics placed a site into the most disturbed category; and (3) all other sites were placed in the intermediate disturbance category. The number of sampled sites within reporting groups by disturbance category is provided in Table 2.

### Variation in disturbance levels for reference sites

Variation in disturbance levels for reference sites across the ecoregion × wetland type reporting groups can have implications for interpreting biological condition. Consequently, we examined overall site-scale disturbance among the least disturbed sites sampled in each reporting group. First, for each of the 10 disturbance indices, we standardized values across all sites to a 0 to 10 continuous scale using the formula: ((observed value − minimum)/(maximum − minimum) × 10). Next, the standardized scores for the 10 indices at each site were summed to obtain an overall disturbance index (DI) value with a potential range from 0 to 100. We evaluated box-and-whisker plots of DI values for the least disturbed sites to identify any differences in reference site quality among reporting groups.

### Vegetation data collection and preparation

Collecting vegetation data at the scale of the conterminous US required specific expertise and training to ensure data quality. Each four-person field crew included a two-member vegetation team, at least one of whom was a botanist with strong expertise in the flora of the state or region where the crew worked. The 53 crews that conducted field work received intensive training in NWCA sampling protocols prior to the 2011 field season (McCauley et al. 2019). Early in the field season, trainers provided on-site feedback to each crew to ensure sampling protocols were correctly and consistently implemented. Crews had access to protocol and logistics support experts who could address questions that arose during sampling throughout the field season. Data from completed vegetation field forms were electronically scanned into the NWCA database, and quality assurance (QA) review was conducted to identify and resolve any potential errors. QA checks included verification that (1) data forms scanned properly, (2) plant names were spelled correctly, (3) data fell into valid ranges and legal values for each data type, and (4) data conformed to a series of logic checks across related data types (USEPA 2016n).

Field and laboratory methods for collecting vegetation data are briefly described here and detailed elsewhere (USEPA 2011a, b). Vegetation data were collected in 2011 during the peak growing season (determined regionally as the time span when most plants were in flower or fruit) to optimize species identification and characterization of species abundance. At each site, data were gathered in five 100-m^2^ vegetation (Veg) plots placed systematically in an AA that was typically a 0.5-ha circular area (Fig. 2). Alternate configurations for AA shape, smaller AA size, and other systematic Veg plot layouts were used only when necessary, as determined by rules related to specific site conditions. All vascular plant taxa occurring in each Veg plot were identified to the lowest taxonomic level possible (typically species or lower, but occasionally to genus or family). Specimens were collected for plant taxa that could not be identified in the field, and were later identified in the lab by regionally designated expert botanists. Taxonomy for all observed vascular plant taxa was standardized to PLANTS Database (USDA-NRCS 2014) nomenclature (USEPA 2016n).

Percent cover for each vascular taxon was estimated, as a direct percentage (0 to 100%), across the entire 100-m^2^ area of each Veg plot (field data: USEPA 2016c). To provide spatial context and facilitate cover estimation, the four quarters of each Veg plot (each quarter representing 25% of the plot area) were demarcated with flagging, and nested quadrats (representing 1% and 10% of the plot area) were established in the SW and NE corners of the Veg plot. Additional data describing trees were collected, including (1) counts in estimated diameter classes for live trees by species and for all standing dead trees and (2) cover estimates for live trees by species within height classes (field data: USEPA 2016g). Data describing percent cover of vascular vegetation in structural classes based on height, cover for non-vascular groups (i.e., bryophytes, lichens, and algae), and ground surface attributes (e.g., water cover and depth; bare ground cover and type; litter cover, depth, and type; and woody debris) were also gathered (field data: USEPA 2016j).

### Candidate metric development and species trait acquisition

Prior to the 2011 survey, potential indicators of wetland condition were identified based on extensive literature review and a collaborative workshop involving approximately 150 wetland scientists and managers (NWCA partners from states, tribes, federal agencies, universities, and other organizations). From this process, key vegetation metric groups, each encompassing a variety of metric types (Table 3), were recognized as biological categories for potential candidate metrics. Among existing wetland VMMIs or VIBIs any given index typically included only a subset of the identified metric groups, and the selected metric groups and metric types varied with wetland type, region, and management objectives (Mack and Kentula 2010). Thus, for the NWCA, we developed numerous candidate metrics for evaluation to identify those that would be broadly responsive to disturbance across multiple ecoregions and wetland types. Plant species data collected in the field were paired with species trait information to develop 405 candidate vegetation metrics of condition (metric data: USEPA 2016h) representing the major metric groups listed in Table 3. All candidate metrics are described in the *NWCA 2011 Technical Report* (USEPA 2016n) and were calculated using scripts written in R Statistical Software, version 3.1.1 (R Core Team 2015). To calculate these 405 candidate vegetation metrics, it was necessary to gather or develop a variety of species trait information (USEPA 2016n).

**Table 3:** Metric groups and component metric types for characterizing vegetation condition

Metric Groups	Major metric types
Taxa composition^a^	Richness, diversity, frequency, cover, and importance for vascular plant species, genera, families, etc.
Floristic quality^a^	Mean coefficient of conservatism, floristic quality assessment index (versions based on species presence, or weighted based on species frequency or cover)
Tolerance/sensitivity to disturbance	Richness and abundance of sensitive, insensitive, tolerant, highly tolerant species
Hydrophytic status^a^	Richness and abundance by wetland indicator status; wetland indices
Life history^a^	Richness and abundance by growth habit type, duration/longevity category, vascular plant category (e.g., ferns, dicots)
Vegetation structure	Frequency, cover, importance, diversity, by structural (height) vegetation groups
Non-vascular	Frequency, cover, importance for ground or arboreal bryophytes or lichens, algae
Ground Surface attributes	Frequency, cover, importance, depth, and types for water, litter, bare ground
Woody debris and snags	Frequency, cover, importance for woody debris, counts for snags
Trees^a^	Richness, counts, or frequency, cover or importance by height or diameter classes

^a^Individual metrics in metric group often included versions based on data describing either all species or native species only. Note: importance metrics combine frequency and cover

Species life history information for growth habit, duration, and plant category was obtained from the PLANTS database (USDA-NRCS 2012) and summarized (see USEPA 2016n) to assign life history traits to the individual vascular taxa observed in the NWCA (trait data: USEPA 2016d). Regionally specific wetland indicator status (obligate, facultative wetland, facultative, facultative upland) for each observed NWCA species based on the National Wetland Plant List (NWPL) (USACE 2014) was downloaded from the PLANTS database (USDA-NRCS 2014), and upland (UPL) status was assigned to all NWCA taxa-region pairs not listed in the NWPL (trait data: USEPA 2016e).

The proportion or abundance of native and nonnative flora at a given location can help inform assessment of ecological condition or stress (Dukes and Mooney 2004; Meyerson and Mooney 2007; Magee et al. 2008, 2010; Ringold et al. 2008). State-level native status (Table 4) was determined for the approximately 13,000 taxa-state pairs observed across the 1138 sampled sites in the conterminous United States (trait data: USEPA 2016b). Native status designations were assigned for each observed taxon-state pair through review of numerous taxonomic and ecological sources (*n* ≈ 85), including state and regional floras and checklists, and state and national floristic databases (USEPA 2016n). For cryptic species or species for which little information was available, consultation with the PLANTS Database (USDA-NRCS 2014) nomenclatural team helped inform native status determinations.

**Table 4:** Definition of state-level native status designations for NWCA taxa

Native status designations	
Native: Indigenous to specific states in conterminous US	
Introduced: Indigenous outside of, and not native in, conterminous US	
Adventive: Native to some areas of the United States, but introduced in location of occurrence	
Alien: Introduced + adventive	
Cryptogenic: Both native and introduced genotypes, varieties, or subspecies Nonnative: Alien + cryptogenic	
Undetermined: Growth forms, families, genera with native and alien species	

Coefficients of conservatism (*C* values, scaled from 0 to 10) describe the responsiveness of individual plant species to disturbance, based on the habitat(s) in which each species typically occurs (Wilhelm and Ladd 1988; Swink and Wilhelm 1979). Lower *C* values reflect greater tolerance and higher values reflect greater sensitivity to disturbance. State-level *C* values for the taxa-state pairs observed during the 2011 NWCA were obtained or developed (USEPA 2016n) by (1) compiling a database of existing state and regionally specific *C* value lists from across the conterminous US, (2) applying the *C* values from this database to the NWCA taxa-state pairs, and (3) identifying the NWCA taxa-state pairs for which *C* values were unavailable. For this latter group, wherever possible, *C* values were assigned based on extension of existing *C* values from ecologically similar states or regions. For a small number of species-state pairs, it was necessary to assign *C* values directly based on habitat preferences and distribution of these taxa. Alien plant taxa were assigned *C* values of 0. The final *C* values for the taxa-state pairs observed in 2011 are provided in a NWCA trait data set (USEPA 2016b).

State or regionally specific *C* values for the suite of individual species occurring at a particular site were used to calculate (1) floristic quality indices and (2) metrics describing sensitivity or tolerance of individual plant species to disturbance (USEPA 2016n). Floristic quality indices and sensitivity or tolerance metrics have been applied as robust indicators of wetland condition in many regions of the US (Lopez and Fennessy 2002; Cohen et al. 2004; Matthews et al. 2005; Bourdaghs et al. 2006; Miller and Wardrop 2006; Milburn et al. 2007; Rocchio 2007; Bried et al. 2013; DeBerry et al. 2015; Deberry and Perry 2015; Gara and Stapanian 2015; Minnesota Pollution Control Agency 2015; Chamberlin and Brooks 2016; Spyreas 2016). Two common floristic quality indices used to describe wetland condition are the *mean coefficient of conservatism* (mean *C*) and the *floristic quality assessment index* (FQAI). Both may be calculated based on species presence only or weighted by species abundance and may include all species or native species only. Plant sensitivity to human-mediated disturbance is often described based on presence or abundance of high *C* value taxa. Conversely, tolerance to disturbance is based on presence or abundance of low *C* value taxa (Mack and Kentula 2010).

### Developing a national-scale vegetation multimetric index

Developing the NWCA VMMI involved three major steps: (1) evaluating candidate vegetation metrics for effectiveness at indicating ecological condition, (2) standardizing (scoring) the best performing metrics, and (3) constructing numerous potential VMMIs from the best candidate metrics and assessing the efficacy of candidate VMMIs to identify the optimum national VMMI. In support of these analyses, vegetation data were divided into calibration and validation data sets (80% and 20% of sampled sites, respectively, Table 2). To encompass the range of disturbance and wetland types represented by the NWCA, the 227 sites for the validation set were designated by stratified-random selection based on both disturbance category (least, *n* = 55; intermediate, *n* = 106; and most disturbed, *n* = 66) and broad wetland type groups (estuarine intertidal (E)): both estuarine herbaceous (EH) and woody (EW), *n* = 69; inland herbaceous: PRLH, *n* = 72; inland woody: PRLW, *n* = 86). Calibration data were used for candidate metric evaluation, metric standardization, and development of potential VMMIs. Validation data were reserved to evaluate the consistency of potential VMMIs to help avoid overfitting to the calibration data. Because all data were collected in 1 year (2011), this comparison does not insure against overfitting to climate conditions present in 2011. However, the performance of the VMMI on the validation data provides the most realistic available indicator of the VMMI’s future performance on new data (Van Sickle 2010), such as the next iteration of the NWCA. A robust potential VMMI using calibration data scoring is expected to similarly distinguish least from most disturbed sites for both calibration and validation data, and we evaluated this ability using box-and-whisker plots and two-sample *t* tests. All analyses related to VMMI development were conducted using R Statistical Software, version 3.1.1 (R Core Team 2015).

#### Step 1—evaluating candidate metrics of condition

Exploratory histograms of values for the 405 candidate vegetation metrics across all 1138 sampled sites (not shown) revealed most were strongly non-normal, thus, nonparametric statistical approaches were used for metric evaluation. We examined the effectiveness of the candidate condition metrics across the spatial scale of the conterminous US (using calibration data, *n* = 911 sites) based on screening criteria for range, repeatability, and responsiveness.

Metrics with narrow range, many zero values, highly skewed distributions, or large numbers of identical values are typically poor indicators of ecological condition that will inadequately detect signals related to disturbance (Stoddard et al. 2008). We developed two tests to define sufficient (PASS) and insufficient (FAIL) range:

1. Identify metrics with a large proportion of 0 values or highly skewed distributions:

- If the 75th percentile = 0, i.e., 75% or more of values are zero, then FAIL
- If the 75th percentile = the minimum OR the 25th percentile = max (indicating 75% of values identical), then FAIL; ensures that majority of values are not the same as the minimum or maximum, and helps eliminate highly skewed variables with mostly a single non-zero value

Test 2. Identify metrics with very narrow ranges:

- If the metric is a percent variable and (max - 25th percentile) < 15%, then FAIL
- If the metric is not a percent variable and (max - 25th percentile) < (max/3), then FAIL

If no component of either range test 1 or 2 resulted in a FAIL, the final assignment for the metric was PASS.

Repeatability was quantified using signal/noise (S:N), which is the ratio of variance in a metric across all sampled sites (signal) to the variance associated with repeat sampling of some or all of the same sites (noise) (Kaufmann et al. 1999). All calibration sites were included in S:N calculation to estimate signal across as wide a gradient as possible, and the 78 revisit sites included in the calibration data (Table 2) that were sampled twice during the field season were used to estimate the noise. S:N for each candidate metric was calculated using the R package “lme4” version 1.1-7 (Bates et al. 2014), with each metric a response variable, SITE_ID (a site identifier) as the main factor in a random effects model, and the variance components from the resulting model used to calculate S:N. Metrics with higher S:N are more likely to show consistent responses to human-caused disturbance, and S:N values ≤ 1 indicate that sampling a site twice yields as much or more metric variability as sampling two different sites (Stoddard et al. 2008). S:N thresholds for retention of metrics have been set in other studies to reflect the variability in the assemblages being sampled, e.g., S:N ≥ 4 or 5 for fish metrics, and 2 for macroinvertebrate metrics (Stoddard et al. 2008). Among the candidate vegetation metrics that passed the range tests (*n* = 329), S:N ranged from 0.9 to 159. We set conservative metric retention criterion for S:N to ≥ 10, or ≥ 5 if a metric type (Table 3) was as yet unrepresented in the suite of metrics passing all other selection criteria.

Responsive candidate metrics distinguish least disturbed (reference) sites from most disturbed sites (Stoddard et al. 2008). To evaluate metric responsiveness, we used a Kruskal-Wallis test (large sample approximation) with thresholds for metric retention at *p* ≤ 0.01 and chi-squared ≥ 10, or ≥ 5 if a metric type was as yet unrepresented in the suite of metrics passing all selection criteria.

To develop a national-scale VMMI, the component metrics need to have wide applicability across wetland types and ecoregions. Only 35 of the initial 405 candidate condition metrics met this criterion by passing all screening filters for range, repeatability, and responsiveness, and these were retained for consideration in VMMI development. This final set of candidate metrics (Table 5) included descriptors of native species composition, floristic quality, sensitivity or tolerance to disturbance, and various life history characteristics. All four metric types included a variety of metrics based on species richness, abundance (cover or frequency), or importance (combining cover and frequency).

**Table 5:** Vegetation metrics that passed range, repeatability, and responsiveness screening filters based on calibration data (*n* = 911 sites)

Metric groups (headings)/Individual metrics (indented)	
Native species	
Percent native richness	
Relative frequency of native species	
Relative cover native species	
Relative importance of native species	
Mean between plot dissimilarity native species	
Floristic quality	
Mean *C* native species	
Mean *C* all species	
Mean *C* native species, cover weighted	
Mean *C* all species, cover weighted	
FQAI native	
FQAI all species	
FQAI native cover weighted	
FQAI all species, cover weighted	
Sensitivity or tolerance	
Richness sensitive species	
Richness tolerant species	
Richness highly tolerant species	
Percent richness sensitive species	
Percent richness tolerant species	
Relative cover sensitive species	
Relative cover tolerant species	
Relative cover highly tolerant species	
Life history	
Percent richness obligate species	
Percent richness facultative wetland species	
Percent richness facultative species	
Wetland index, cover weighted	
Percent richness native graminoid species	
Relative cover native graminoid species	
Percent richness native monocot species	
Relative cover native monocot species	
Percent richness native herbaceous species	
Relative cover native herbaceous species	
Richness vine species	
Percent richness annual species	
Percent richness perennial native species	
Other	
Mean litter depth	

Metric formulas defined in (USEPA 2016n)

#### Step 2—metric scoring

The final 35 candidate metrics were standardized on a 0 to 10 continuous scale using the calibration data. The metrics were scored based on interpolation of metric values between the 5th (floor) and 95th (ceiling) percentiles across all calibration sites (Blocksom 2003). The direction of each metric was determined by the direction of the difference between the mean of the least disturbed sites and the mean of the most disturbed sites. If the difference was positive, better condition is associated with higher metric values, and if negative, the reverse is true. For metrics decreasing with increasing disturbance, the ceiling was scored as 10 and the floor as zero. Conversely, for metrics that increased with increasing disturbance, the floor was scored as 10 and the ceiling as zero. Scores were truncated to 0 or 10 if observed values fell outside the floor to ceiling range. The metric scoring based on the calibration data was applied to the validation data.

#### Step 3—generating and screening candidate VMMIs

Determining the optimal set of metrics for inclusion in an MMI is a complex process. For example, using MMIs describing stream assemblages of macroinvertebrates and fish as a test case, Van Sickle (2010) demonstrated that combining the set of maximally responsive individual candidate condition metrics was unlikely to yield the MMI with the best performance; that is, “the performances of individual metrics do not reliably predict the joint performance of their summed index.” He recommended comparing the performance of multiple MMIs built from all possible (or a large number of random) metric subsets to obtain a shortlist of high-performing MMIs. A single best MMI could then be selected from this list based on its overall performance and attributes of its individual metrics (e.g., interpretability, reliability, applicability across diverse environmental conditions).

We also found in preliminary analyses of NWCA data that candidate VMMIs based on various sets of the highest performing metrics, did not perform as well as the best VMMIs built using Van Sickle’s approach (USEPA 2016n). Accordingly, we adapted Van Sickle’s methods (2010) to develop numerous candidate national-scale VMMIs for the NWCA. This randomization procedure allowed determination of an optimum number of metrics for inclusion in a VMMI and objective calculation and evaluation of many thousands of potential VMMIs. Candidate VMMIs were developed based on all sites in the calibration data set (*n* = 911) and the 35 final scored candidate metrics. All candidate metrics were considered in creating the random combinations of metrics for the candidate VMMIs; there was no requirement for representing all metric groups (Table 5) in each candidate VMMI. Each potential VMMI was calculated and placed on a 100-point scale using the formula: VMMI = *Σ* metric scores × 10/number of metrics.

The most parsimonious number of metrics to include in a national VMMI was identified by examining numerous candidate VMMIs based on metric-sets of various sizes (4, 6, 8, and 10 metrics). We selected a random set of 10 metrics, then randomly selected 8 metrics from that set of 10. A set of 6 metrics was randomly selected from the 8 metric set, and a set of 4 was randomly selected from the 6 metric set. This process was repeated 5000 times for combinations of 4, 6, 8, and 10 metrics, for a total of 20,000 potential VMMIs. The resulting potential VMMIs were then evaluated using a series of performance criteria to determine which were most effective. Parametric statistics were used because MMIs that are the sum of several metrics tend to have normal distributions (Fore et al. 1994), even though individual metrics may be skewed.

Performance statistics for evaluating the candidate VMMIs included measures of redundancy, sensitivity, repeatability, and precision. To avoid metric redundancy, it has been generally argued that metrics included in a MMI should not be strongly correlated (e.g., Cao et al. 2007; Stoddard et al. 2008; Pont et al. 2009; Van Sickle 2010). In addition, Van Sickle (2010) demonstrated that smaller mean correlation among component metrics resulted in stronger performing MMIs. Thus, only candidate VMMIs with maximum and mean Pearson correlations among component metrics of < 0.75 and < 0.5, respectively, were retained for further review. Next, we evaluated the sensitivity of each VMMI using an interval test (Kilgour et al. 1998) to determine the percentage of most disturbed sites that were identified as having impacted vegetation, based on the VMMI. The interval test was used to determine whether the VMMI value for a given site was significantly lower than the 5th percentile of the reference VMMI distribution. The test assumed a normal reference distribution for the VMMI and used a non-central *F* distribution to model uncertainty in the 5th percentile of that distribution, which was necessary because the estimate of the 5th percentile was based on a finite sample from the reference population (Van Sickle 2010). This test allowed us to compare an individual sample to a critical *F* value to determine whether impact on vegetation was detected. It is a more conservative approach than simply comparing the VMMI at a site to a 5th percentile threshold because it takes into account that uncertainty around the estimate of the 5th percentile (Kilgour et al. 1998). Repeatability for each candidate VMMI was assessed within the calibration data using a S:N ratio (Kaufmann et al. 1999) calculated based on data from the primary sampling visits for each site (*n* = 911 sites) and repeat sampling visits for a subset of these sites (*n* = 78 revisit sites). The standard deviation (SD) of VMMI values among the least disturbed reference sites was used to describe precision.

To identify the most effective candidate VMMIs in each metric set (4-, 6-, 8-, or 10-metric VMMIs), we first arranged all candidate VMMIs that passed the correlation filter in order of decreasing sensitivity. Typically, the VMMIs with the lowest correlations were also the most sensitive. Next, for the one or two hundred most sensitive VMMIs in each set, those with the lowest mean and maximum correlation among component metrics were identified. Among these, the VMMIs with the highest S:N and smallest SD were examined. We found the highest performing national-scale candidate VMMIs from across all the metric size sets included 4 metrics. Some 6-metric VMMIs also performed well, but typically included multiple metrics that reflected information contained in a single metric in the best performing 4-metric VMMIs. Few 8- or 10-metric VMMIs performed well, and all had lower sensitivity and higher metric correlations than the best VMMIs based on fewer metrics. Thus, the most parsimonious VMMI would have between 4 and 6 metrics.

Based on these screening results, we examined a larger pool of potential VMMIs based on 4, 5, or 6 metrics to identify the best overall national VMMI. Candidate VMMIs using all combinations of 4 (*n* = 52,360) and 5 (*n* = 324,632) metrics were generated. We did not try to construct VMMIs for all combinations of 6 metrics (*n* = 1,623,160) because the computing time required was problematic; however, we evaluated 400,000 randomly assembled 6-metric VMMIs. From this set of analyses, potential VMMIs passing the mean and maximum correlation filters included: 578 with 4 metrics, 483 with 5 metrics, and 202 with 6 metrics. This group of candidate VMMIs was further evaluated based on sensitivity, repeatability, and precision to identify the highest performing VMMI in each of the three metric size sets. Finally, box-and-whisker plots were created for the most promising 4-, 5-, and 6-metric VMMIs to evaluate responsiveness by comparing how well each VMMI distinguished the least and most disturbed sites for (1) calibration data vs. validation data at the national-scale and (2) for each of the 10 NWCA reporting groups (Table 2). All of this information was viewed together to inform the selection of the final national VMMI. For the selected VMMI, a final evaluation of responsiveness was conducted using two-sample *t* tests to compare mean VMMI values between all sampled least and most disturbed sites by reporting group.

### Condition category definition and wetland area estimation

Biological condition categories (good, fair, and poor) were defined in relation to reference, following the percentile approach as described in Paulsen et al. (2008), which is based on the distribution of observed values for the final VMMI across all sampled least disturbed sites (both calibration and validation). Condition thresholds were defined separately for each of the 10 reporting groups to account for natural variation among ecoregions and wetland types and for differences in disturbance levels across the conterminous US. *Good* condition was defined by VMMI values greater than or equal to the 25th percentile, *fair* condition ranged from the 5th up to the 25th percentile, and *poor* condition was delimited as less than the 5th percentile for the least disturbed sites. Each NWCA site was assigned to a condition category based on its VMMI value and the thresholds for the reporting group in which the site occurred.

Condition status and the sample-weights for the sampled probability sites (*n* = 967) were used to estimate the area of the inference (sampled) population in good, fair, and poor condition at the national scale and for each of the ten reporting groups (USEPA 2016m, n). Although the condition status for individual sites is determined by VMMI thresholds for condition categories specific to the NWCA reporting group in which the site occurs, condition estimates can also be summarized for other wetland subpopulations, assuming a sufficient number of sample sites (ideally ≥ 50) (Larsen 1997). Using this approach, we also examined estimates of wetland area by condition categories for xeric vs. mesic/montane landscapes in the West. In all cases, estimates and confidence intervals for area in good, fair, and poor condition were calculated using the R package “spsurvey” (Kincaid and Olsen 2016).

## Results and discussion

### Disturbance and reference site quality

**Fig. 3:**
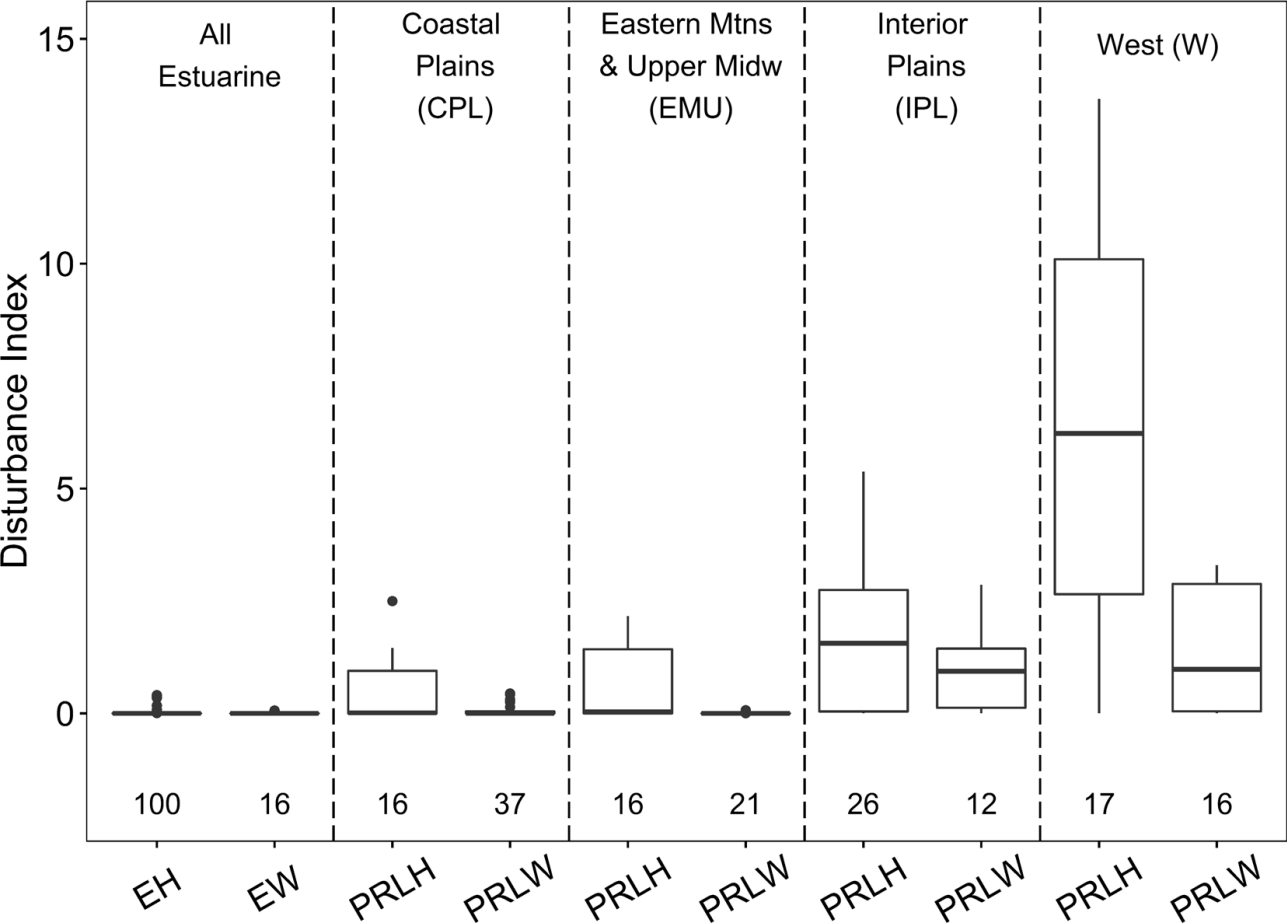
Comparison of reference site quality by NWCA reporting group (see Table 2), based on the overall site-scale disturbance index (DI) for the least disturbed sites. Higher DI values represent greater site-scale environmental disturbance. For each boxplot, the box is the interquartile (IQR) range, line in the box is the median, and whiskers represent the most extreme point a distance of no more than 1.5 × IQR from the box. Values beyond this distance are outliers

The site-level disturbance index (DI) ranged from 0 to 37 across all sampled sites (*n* = 1138) and from 0 to 14 for sampled reference sites (*n* = 277). Reference quality, as reflected by DI values for the least disturbed sites, varied by reporting group (Fig. 3). Reference sites in the estuarine intertidal reporting groups (EH and EW) had the lowest observed site-scale disturbance. Among inland wetland reference sites within ecoregions, the herbaceous (PRLH) types tended to have greater or more variable levels of disturbance than did the woody (PRLW) types. In addition, for inland wetland types, the quality of sampled reference sites was better (median DI was lower) and less variable (interquartile ranges smaller) for ecoregions in the eastern half of the country (CPL, Coastal Plains; EMU, Eastern Mountains and Upper Midwest) than in the western half (IPL, Interior Plains; W, West). Regional variation in reference site quality has been observed for other aquatic systems in NARS assessments (e.g., Herlihy et al. 2008; USEPA 2016k) and is likely to occur in most large-scale studies because disturbance intensity and human land uses often vary geographically (Stoddard et al. 2006).

### The national-scale VMMI

**Fig. 4:**
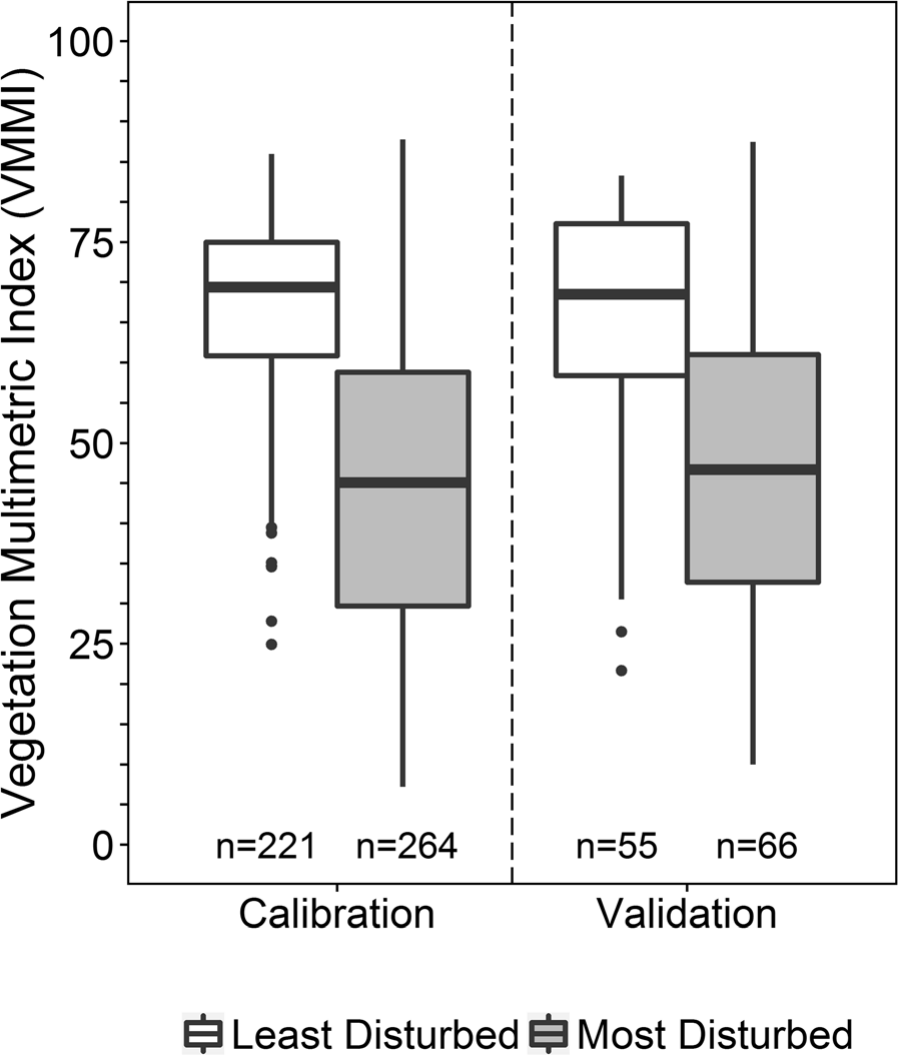
Comparison of the NWCA national vegetation multimetric (4-metric) index (VMMI) for the calibration and validation data sets, contrasting all sampled least and most disturbed sites in each data set. Higher VMMI values reflect better biological condition. Boxplots: box is interquartile (IQR) range, line in the box is the median, and whiskers represent most extreme point a distance of no more than 1.5 × IQR from the box. Values beyond this distance are outliers. Numbers below each boxplot represent number of the least disturbed or most disturbed sites sampled

**Fig. 5:**
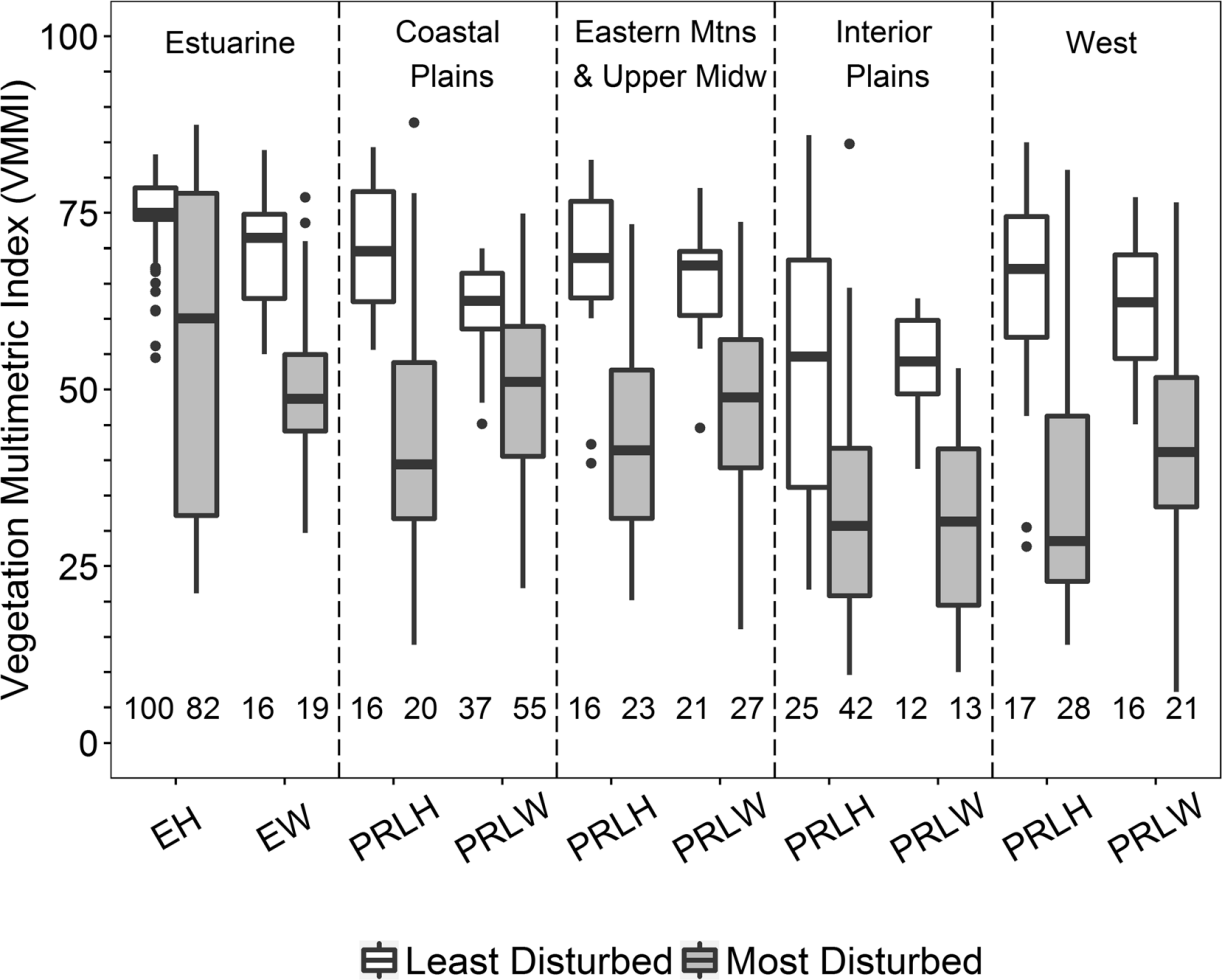
Comparison of the NWCA national vegetation multimetric (4-metric) index (VMMI) values for all sampled least and most disturbed sites (both calibration and validation) by reporting group (see Table 2). Higher VMMI values reflect better biological condition. Boxplots: box is interquartile (IQR) range, line is the median, and whiskers represent most extreme point at a distance no more than 1.5 × IQR from box. Values beyond whiskers are outliers. Numbers below boxplots are numbers of the least or most disturbed sites sampled in each reporting group

Among the 4-, 5-, and 6-metric candidate VMMIs that passed the between-metric correlation screens, the VMMI in each metric-size group with the greatest sensitivity was also characterized by component metrics that were relatively easy to measure and interpret (Table 6). These three top-performing VMMIs shared the same sensitivity value (48.1%) and had similar standard deviation (11.2 to 12.5) in reference site VMMI values. Repeatability (S:N) for all three indices was high, ranging from 16.0 to 23.4, with S:N for the 4-metric VMMI in the middle at 19. However, compared to the 5- and 6-metric VMMIs, the 4-metric VMMI had lower maximum and mean correlation among metrics. The 4-metric VMMI included a floristic quality assessment index (FQAI) based on all species present, relative importance of native species, richness of disturbance-tolerant species, and relative cover of native monocot species (Table 7). Three of these metrics (FQAI, richness of disturbance-tolerant species, and relative cover of native monocots) were also components of the 5- and 6-metric VMMIs (Table 6). The two additional metrics incorporated in the 5-metric VMMI (native species percent richness and relative cover), when considered together, provide information similar to the relative importance of native species metric of the 4-metric VMMI. Likewise, two metrics included in the 6-metric VMMI (relative frequency and relative cover of native species) are the specific components of the relative importance of native species metric. The final metric making-up the 6-metric VMMI was the mean coefficient of conservatism (mean *C*), which, like FQAI, describes overall floristic quality. Similar sensitivity for the top three candidate VMMIs was not surprising given their overlap in ecological content. However, obtaining the same sensitivity value for all three was likely somewhat coincidental; for each of the best VMMIs, 128 most disturbed sites tested as below the 5th percentile of VMMI values for the least disturbed sites but 18 sites varied as impacted or not with no particular pattern across the best 4-, best 5-, and best 6-metric VMMIs. Box-and-whisker plot comparisons of VMMI values across least and most disturbed sites, nationally and by reporting group (shown only for the 4-metric VMMI; Figs. 4 and 5) revealed similar distributions for the 4-, 5-, and 6-metric indices. However, the 4-metric VMMI exhibited slightly greater separation between the least and most disturbed sites for the IPL-PRLH and the W-PRLH reporting groups (Fig. 5), where disturbance levels among reference sites were relatively high and DI values most variable (Fig. 3).

**Table 6:** The highest performing 4-, 5-, and 6-metric VMMIs developed using calibration data (n = 911 sites)

VMMI	Metrics for candidate VMMI	L site mean	L site SD	S:N	Max *r* among metrics	Mean *r* among metrics	Sensitivity (%)
4-metric	Floristic quality assessment index Relative importance native species Richness disturbance-tolerant species Relative cover native monocots	67.3	11.5	19.4	0.396	0.101	48.1
5-metric	Floristic quality assessment index Percent richness native species Relative cover native species Richness disturbance-tolerant species Relative cover native monocots	71.6	11.2	16.0	0.599	0.195	48.1
6-metric	Floristic quality assessment index Relative frequency native species Relative cover native species Richness disturbance-tolerant species Relative cover native monocots Mean coefficient of conservatism	72.1	12.5	23.4	0.727	0.306	48.1

L = least disturbed (reference) sites, *n* = 222; M = most disturbed sites, *n* = 266. SD = standard deviation, S:N = signal/noise (based on the 911 sampled sites and 78 revisit sites from calibration data set), *r* = Pearson correlation. Sensitivity = Percent M sites with VMMI values significantly less than the fifth percentile of the distribution of VMMI values for L sites based on an interval test, alpha = 0.05 (Kilgour et al. 1998; Van Sickle 2010)

**Table 7:** The four metrics included in the final NWCA vegetation multimetric index (VMMI)

Metric name	Metric description	Calculation^a^
Floristic quality assessment index (FQAI)	Based on all species observed	$FQAI=\sum C{C}_{ij}/\sqrt{N_j}$ where CC*ij* = coefficient of conservatism for each unique species *i* at site *j*, N = number of species at site *j*
Relative importance native species	Combines relative cover and relative frequency for native taxa at each site	((∑ Absolute Cover native species_*i*_ /∑ Absolute Cover all species_*i*_) X 100 + (∑ Frequency native species_*i*_ /∑ Frequency all species_*i*_) X 100 )/2 where for each unique species *i*: Absolute Cover = 0-100%, Frequency = 0-100%, calculated as the percent of Veg Plots in which it occurred
Richness disturbance tolerant species	Tolerance to disturbance defined as coefficient of conservatism (CC) ≤ 4	Number of taxa with CC ≤ 4 occurring at a site
Relative cover native monocots	Relative cover of native monocot species at each site	(∑ Absolute Cover native monocot species_*i*_ /∑ Absolute Cover all species_*i*_) X 100

^a^Calculation of metrics is based on data collected in the five 100-m^2^ vegetation plots at each site

Based on the overall performance of the 4-metric VMMI and parallel content in the 5- and 6-metric VMMIs, we selected the 4-metric index (Table 7) as the most parsimonious and adopted it as the national-scale VMMI for the 2011 NWCA. The floor and ceiling values and formulas for standardizing the four metrics comprising the national VMMI are provided in Table 8. Standardized metric scores range continuously from 0 to 10, with higher values reflecting more intact conditions. The VMMI for each sampled site was calculated by summing the scored values for the four metrics and multiplying by a scaling factor (10/4) to place it on a continuous 0 to 100 scale.

**Table 8:** Floor and ceiling values, disturbance response, and interpolation formula for scoring final VMMI metrics. Final scores for each metric increase with disturbance

Metric	Raw data response to disturbance	Floor (5th percentile)	Ceiling (95th percentile)	Scoring formula (observed = metric value at a site)
Floristic quality assessment index	Decreases	6.94	38.59	(Observed–6.94)/(38.59–6.94) × 10
Relative importance native species	Decreases	44.34	100	(Observed–44.34)/(100–44.34) × 10
Richness disturbance-tolerant Species	Increases^a^	0	40.0	(40–Observed)/(40–0) × 10
Relative cover native monocots	Decreases	0.06	100	(Observed–0.06)/(100–0.06) × 10

Scoring based on calibration data (*n* = 911 sites) and applied to all data (*n* = 1138 sites)

^a^Scoring reversed for metrics where raw data increases with disturbance. Scores truncated to 0 or 10 if observed values fell outside the floor to ceiling range

Box-and-whisker plot comparisons of the 4-metric VMMI values for the least and most disturbed sites within the calibration and the validation data sets showed similar patterns in the distribution of VMMI values, and clear separation of least disturbed from most disturbed sites in both data sets (Fig. 4). However, not surprisingly, the box-and-whisker plots did indicate somewhat degraded responsiveness of the VMMI in distinguishing between least and most disturbed sites for the validation data relative to the calibration data. To examine the severity of this degradation, we conducted a two-sample *t* test comparing VMMI means between the least and most disturbed sites for the calibration data and for the validation data (Table 9). The VMMI means across the two data sets were similar for the least disturbed and for the most disturbed sites, though standard errors were somewhat higher for the validation data where sample sizes were smaller. For both data sets, VMMI values for the least and most disturbed sites were highly significantly different (*p* < 0.0001), though the *t* statistic was smaller for the validation data. Because the mean VMMI values were similar and the 4-metric VMMI significantly distinguishes reference from most disturbed for both calibration and validation data, we concluded that it is likely to be a satisfactory indicator of condition. This consistency is promising and hopefully presages reliable VMMI results for data collected in future years.

**Table 9:** Comparison of vegetation multimetric index (VMMI) means between the least and most disturbed sites for calibration and validation data sets using two-sample *t* tests

	Least disturbed	Most disturbed			
Dataset	Mean ± SE	Mean ± SE	*t* statistic	df	*p* value
Calibration	67.32 ± 0.77	45.72 ± 1.18	15.33	443.79	<0.0001
Validation	65.76 ± 1.99	47.03 ± 2.44	5.94	117.52	<0.0001

See Table 2 for sample sizes of the least and most disturbed sites

*SE* standard error, *df* degrees of freedom based on Welch approximation because variances between the least and most disturbed sites are unequal

Next, we looked at the performance statistics for the national VMMI across all sampled sites. S:N was high (20.9, *n* = 1138 sites), reflecting consistency between repeat samplings during the same field season. The low maximum (0.4) and mean (0.1) correlation values indicate that individual metrics contribute unique information about condition. Sensitivity based on calibration sites (*n* = 911) was 48.1%, but 42.8% when all sampled sites (*n* = 1138 sites) were considered. This difference is likely related to increased variability in reference site quality with the addition of the validation data and consequently increased variability in VMMI values among the least disturbed sites (e.g., SD: calibration = 11.5, all sites = 12.2). Interval test results are influenced by SD, so within-group VMMI values with more variation among least disturbed sites will tend to result in more most disturbed sites inside the reference range (Van Sickle 2010). Nevertheless, the sensitivity levels observed for the national VMMI for the 2011 NWCA compare favorably with sensitivity values reported for MMIs for other biological assemblages (e.g., wadeable stream MMIs for macroinvertebrates, vertebrates, or fish where sensitivity for 6 MMIs ranged from ~ 10 to 40% and for 2 MMIs was ~ 60% (Van Sickle 2010)).

VMMI values for the least disturbed sites varied in median and range across reporting groups (Fig. 5), reflecting differences in plant species composition and abundance across the NWCA ecoregions and wetland types (see Fig. 2 in Herlihy et al. 2019a). To address this natural variability, threshold values for good, fair, and poor condition were set within reporting groups based on the distribution of the VMMI values for the least disturbed sites within each group (Table 10). In addition, disturbance levels, and, therefore, the quality of reference sites, varied ecoregionally and by wetland type (Fig. 3). This covariation of natural vegetation and disturbance with ecoregion and wetland type means that the VMMI thresholds reflect variation both in natural conditions and in the quality of reference sites in each NWCA reporting group. As a result, reporting group differences in level of disturbance for sampled reference sites have implications for interpreting and making comparisons between good, fair, and poor condition status. Groups with poorer quality reference sites will have a lower bar for defining good condition than groups with higher quality reference sites (Paulsen et al. 2008; Herlihy et al. 2019a).

**Table 10:** Condition thresholds for each NWCA reporting group based on vegetation multimetric index (VMMI) values

NWCA reporting group	Poor condition VMMI threshold	Good condition VMMI threshold
ALL-EH	˂ 65.0	≥ 74.1
ALL-EW	˂ 56.0	≥ 62.9
CPL-PRLH	˂ 57.3	≥ 62.5
CPL-PRLW	˂ 52.8	≥ 58.6
EMU-PRLH	˂ 41.6	≥ 63.0
EMU-PRLW	˂ 55.8	≥ 60.5
IPL-PRLH	˂ 25.3	≥ 36.2
IPL-PRLW	˂ 40.3	≥ 49.4
W-PRLH	˂ 30.0	≥ 57.4
W-PRLW	˂ 47.9	≥ 54.4

See the “Methods” section for threshold criteria. Sites falling between the good and poor thresholds are considered fair. NWCA reporting groups are defined in Table 2

Nevertheless, the ability of the VMMI to distinguish reference from most disturbed sites was highly significant for all 10 reporting groups (Table 11), and box-and-whisker plot comparisons (Fig. 5) indicated clear separation of VMMI values based on interquartile ranges for the least and most disturbed sites for 8 of the 10 groups. For the estuarine intertidal herbaceous (ALL-EH) and the Interior Plains inland herbaceous (IPL-PRLH) wetland groups, the distinction between VMMI values for reference and most disturbed sites was somewhat less pronounced (Fig. 5), although still strongly significant (Table 11).

**Table 11:** Comparison of vegetation multimetric index (VMMI) means between least and most disturbed sites by reporting group using two-sample *t* tests

Reporting group^a^	*t* statistic	*p* value	df
ALL-EH	8.24	< 0.00001	180
ALL-EW	4.56	0.00007	33
CPL-PRLH	4.39	0.0001	34
CPL-PRLW	5.45	< 0.00001	90
EMU-PRLH	5.53	< 0.00001	38
EMU-PRLW	4.84	0.00002	46
IPL-PRLH	4.52	0.00003	66
IPL-PRLW	4.90	0.00005	24
W-PRLH	4.96	0.00001	43
W-PRLW	4.08	0.00025	35

*df* degrees of freedom

^a^Definitions and sample sizes provided in Table 2

VMMI values observed in reference sites of the ALL-EH group overlapped slightly with the upper end of VMMI values for the most disturbed sites (Fig. 5). This overlap in vegetation condition may relate to disturbance patterns in the ALL-EH groups. Most (91 of 100) of the least disturbed EH sites were minimally disturbed, that is, had zero values for all 10 disturbance indices used in assigning disturbance status (Herlihy et al. 2019a). Concurrently, the most disturbed EH sites (*n* = 82) spanned a gradient from relatively low to relatively high disturbance (DI range = 2.2 to 28, mean ± SD= 9 ± 5). In addition, all but two of the EH reference sites were located in the CPL, while the most disturbed sites spanned greater ecoregional variation (CPL = 55; EMU = 7; W = 20).

In contrast, the overlap in VMMI values between reference and most disturbed sites in the IPL-PRLH group (Fig. 5) indicates a wide range in vegetation condition across least disturbed sites. This wide variation in VMMI values for IPL-PRLH reference sites was likely related, in part, to differences in natural environmental conditions across this large diverse region (IPL, Fig. 1), and, in part, to reference site quality in the Interior Plains as reflected by the DI (Fig. 3). Moreover, agricultural land use (landscape-scale, 1000 m area surrounding the AA) was greatest for NWCA sampled sites in the Interior Plains (IPL % cover agriculture, ≥ 10% for 82% of sites; median ≈ 50%) (Herlihy et al. 2019c), and this could also relate to the observed range in VMMI values among least disturbed sites for the IPL-PRLH group.

Taken together, the combined performance results for the final VMMI support its utility for assessing wetland biological condition at a national scale. We also found, in other work, that the national VMMI with reporting group thresholds for condition categories performed as well or better than top-performing candidate VMMIs based on wetland type groups: estuarine intertidal (EH + EW), inland herbaceous (PRLH), or inland woody (PRLW) (USEPA 2016n). Moreover, because the national VMMI is based on the same set of widely applicable component metrics, it standardizes assessment and provides a consistent context for describing wetland condition at the continental-scale of the conterminous US or for large wetland subpopulations. Site-level VMMI values, condition status, component metric values, and component metric scores are available in USEPA (2016i).

### Wetland biological condition

**Fig. 6:**
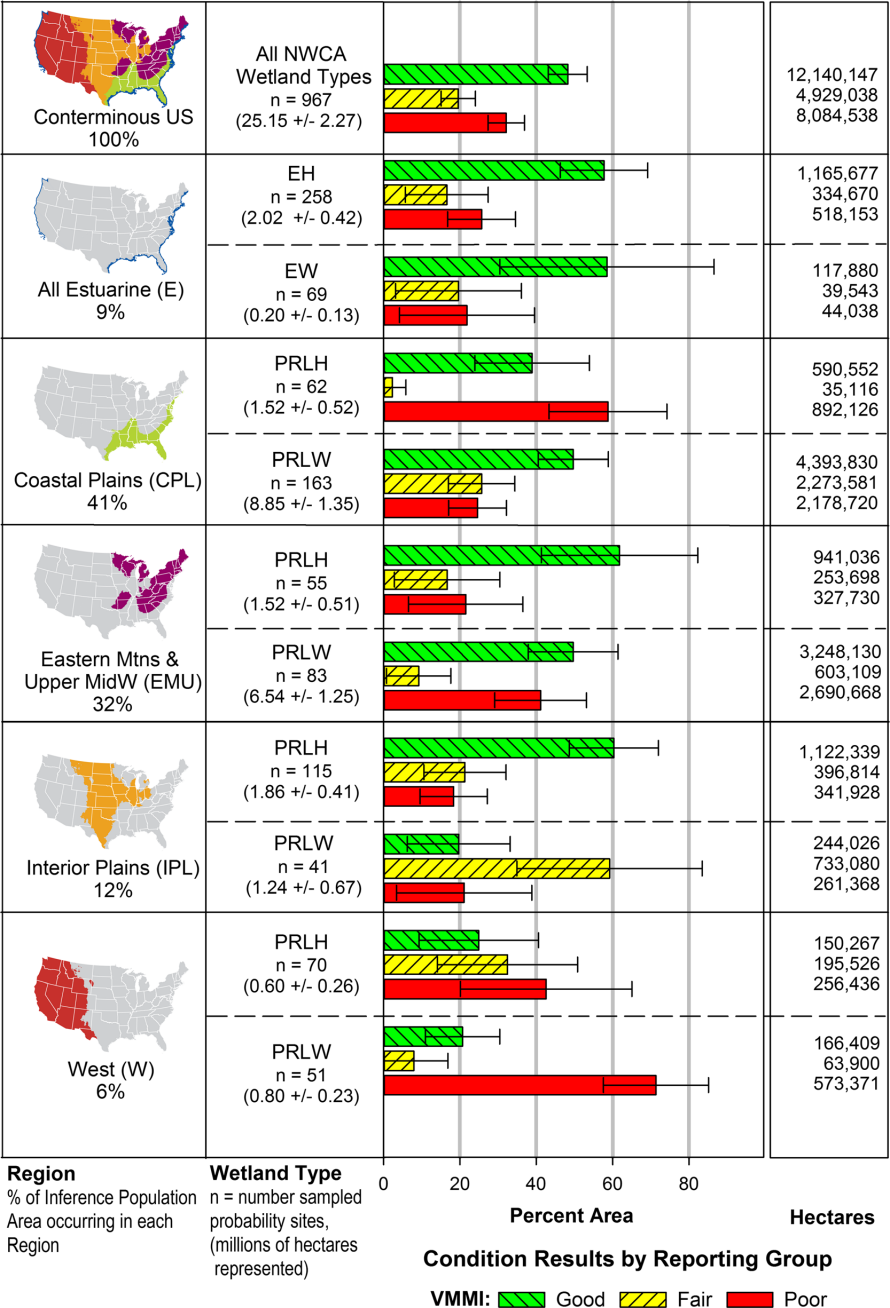
Estimates of wetland area and area in good, fair, and poor conditions based on the vegetation multimetric index (VMMI), for the inference population (25.15 ± 2.27 million hectares) represented by the NWCA sampled probability sites (*n* = 967). Results are reported nationally and by reporting group (region (micro maps) by wetland type (prefix E = estuarine intertidal wetlands, prefix PRL = inland wetlands (palustrine, riverine, or lacustrine), suffix H = herbaceous, suffix W = woody)). Sampled probability sites and sample-weights from the survey design were used to estimate areas. Margin of error estimates and error bars are two-sided 95% confidence intervals

Total wetland area and the area in good, fair, and poor biological condition as indicated by the national VMMI, for the 2011 NWCA inference or sampled population (represented by the 967 sampled probability sites) were estimated nationally and for the 10 reporting groups as hectares and percent area including 95% confidence intervals (Fig. 6). The 25.15 ± 2.27 million hectares of wetland across the conterminous US, represented by the sampled population, are unequally distributed among wetland types and ecoregions, reflecting the spatial distribution of wetlands nationally (Olsen et al. 2019). Estuarine intertidal systems made-up about 9% (EH ≈ 8%, EW ≈ 1%) of this area. A much larger proportion of the sampled population was inland wetland with area varying by ecoregion and wetland type: 41% in the Coastal Plain (CPL-PRLH ≈ 6%, CPL-PRLW ≈ 35%), 32% in the Eastern Mountains and Upper Midwest (EMU-PRLH ≈ 6%, EMU-PRLW ≈ 26%), 12% in the Interior Plains (IPL-PRLH ≈ 7%, IPL-PRLW ≈ 5%), and only 6% in the West (W-PRLH ≈ 3%, W-PRLW ≈ 3%). The inference population represented 65.5% (± 3.9%) of the estimated area of the NWCA target population, with the percent of target population area sampled varying across the 10 reporting groups primarily in relation to patterns of landowner denial for site access or to physical inaccessibility of a site (Olsen et al. 2019). At the ecoregion scale (Table 12), the inference population represented the greatest percentage of the target population area in the Eastern Mountains and Upper Midwest (80 ± 8.3%), and the least in the West (40 ± 8.5%).

**Table 12:** Estimated wetland area in NWCA target population and percent of target population represented by inference (sampled) population by NWCA ecoregion and for two sub-regions (XER, WMT) of the West (W)

Ecoregion	Estimated target population area (millions of ha)	% target population area represented by inference population
CPL	19.70 ± 1.51	63 ± 5.7
EMU	10.01 ± 1.16	80 ± 8.3
IPL	4.97 ± 0.74	62 ± 7.7
W	3.73 ± 0.59	40 ± 8.5
XER	2.08 ± 0.18	52 ± 9.5
WMT	1.65 ± 0.29	24 ± 10.1

**Fig. 7:**
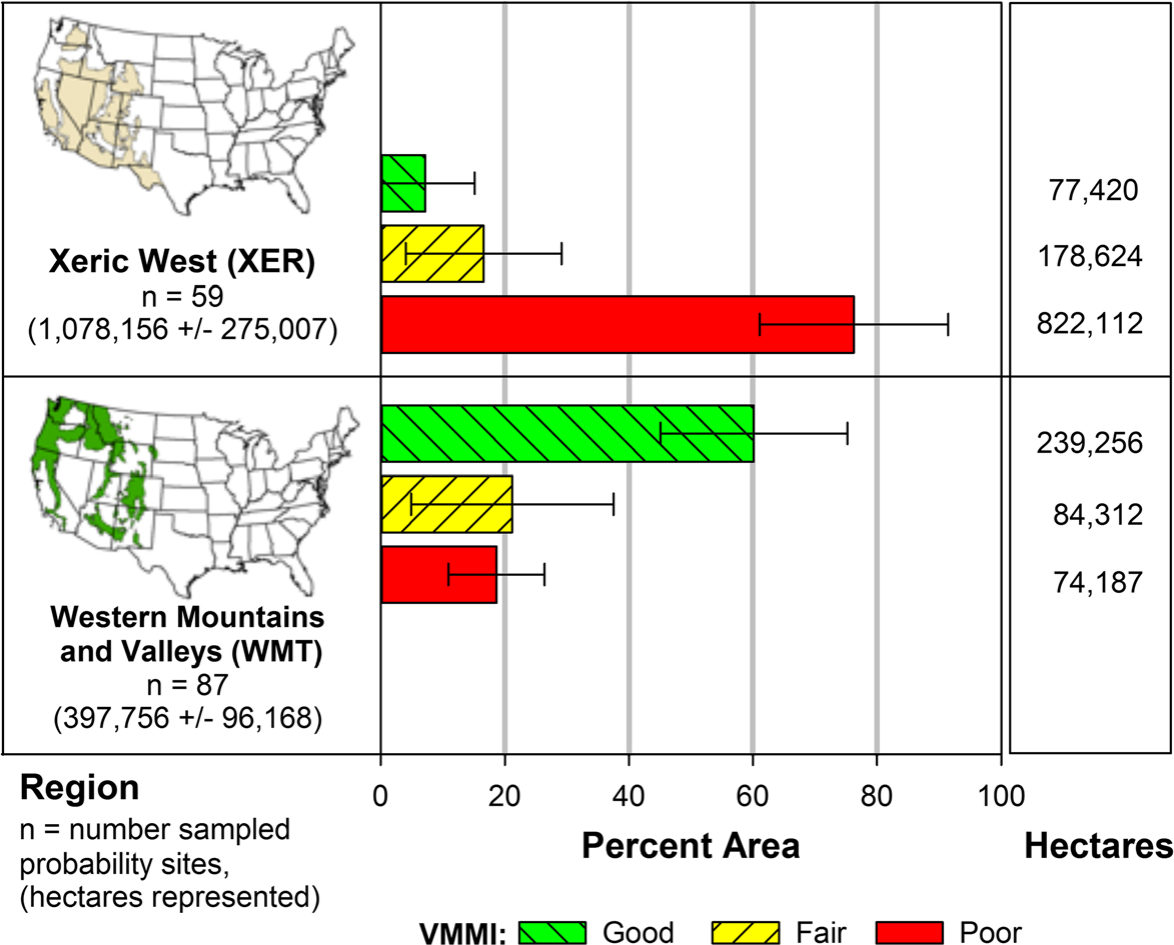
Estimates of wetland area and area in good, fair, and poor conditions based on the vegetation multimetric index (VMMI), for the inference subpopulation represented by NWCA sampled probability sites in the Xeric West (XER) and in the Western Mountains and Valleys (WMT). Both regions included estuarine herbaceous (EH), inland herbaceous (PRLH), and inland woody (PRLW) wetland types (defined in Table 2). Area estimates were completed using sampled probability sites and their sample-weights from the survey design. Margin of error estimates and error bars are two-sided 95% confidence intervals

Margin of error estimates are two-side 95% confidence intervals. See Figs. 1 and 7 for ecoregion definitions

Nationally, less than half of the evaluated wetland resource was in good biological condition: 48 ± 5.1% was estimated to be in good, 20 ± 4.5% in fair, and 32 ± 4.8% in poor condition (Fig. 6). The concentration of wetland area in the eastern half of the country means national-scale condition patterns are strongly influenced by patterns observed in the CPL and EMU ecoregions. However, condition results varied among the 10 reporting groups, providing information about level of impact and potential vulnerabilities at regional and wetland type scales. Estuarine intertidal herbaceous (ALL-EH) and woody (ALL-EW) wetland types shared similar patterns in condition; with 58% of their respective area in good condition and approximately 42% in both types in fair or poor condition (though the margin of error for ALL-EW was greater). In the Coastal Plains, inland herbaceous systems appeared to have lower biological condition than inland woody systems. The percent area in good condition was somewhat less for CPL-PRLH (39 ± 15%) compared to CPL-PRLW (50 ± 9%), but the percent area in poor condition was substantially greater in CPL-PRLH (59 ± 15%) than in CPL-PRLW (25 ± 8%). In contrast to the CPL, differences in condition patterns between PRLH and PRLW in the Eastern Mountains and Upper Midwest were less pronounced. However, EMU had a somewhat greater percent area in poor condition for woody (EMU-PRLW = 41 ± 12%) vs. herbaceous (EMU-PRLH = 21 ± 15%) systems.

When considering condition estimates in the Interior Plains and West (Fig. 6), it is important to recall that reference site quality based on DI values for least disturbed sites (Fig. 3) was lower for inland wetlands in the IPL and W than in the eastern half of the conterminous US. Thus, the lower bounds for good condition for IPL and W inland wetland reporting groups (Table 10) reflect not only reporting group-specific biotic attributes but also the greater disturbance observed in reference sites, potentially setting a lower standard for good condition than in eastern reporting groups. IPL herbaceous wetland also exhibited a wide range in VMMI values for least disturbed sites (Fig. 5). Consequently, the 60 ± 12% of IPL-PRLH wetland area categorized as having good vegetation condition must be viewed in the context of variability in reference sites for this reporting group. In contrast, in the IPL-PRLW, the sampled least disturbed sites exhibited a smaller range in DI (Fig. 3) and less variability in VMMI values (Fig. 5) than the IPL-PRLH. The majority of the inland woody (IPL-PRLW) area was estimated to be in fair condition (59 ± 24%).

The greatest disturbance among sampled reference sites, based on the DI, was in the West, particularly for herbaceous inland systems (Fig. 3). Estimates of biological condition (Fig. 6) for W-PRLH showed the majority of its area in poor (43 ± 22%) to fair (32 ± 18%) condition, with only 25 ± 16% in good condition, but note the overlapping margins of error. Inland woody (W-PRLW) types appeared to be the most highly impacted of any reporting group, with estimates showing only 21 ± 10% of their area in good condition and the greatest proportion in poor condition (71 ± 14%). Wetlands in the West are represented by diverse plant communities and span abiotic environments ranging from xeric to mesic to montane. For VMMI development in the 2011 NWCA, it was necessary to merge this varied landscape into one aggregated ecoregion (Fig. 1) because of sample size limitations within aggregated wetland types (Herlihy et al. 2019a). However, other NARS focusing on stream ecosystems divided the West into two aggregated ecoregions, the Xeric West (XER) and the Western Mountains and Valleys (WMT) (Paulsen et al. 2008).

To evaluate whether patterns of biological condition varied across the xeric to mesic/montane west, we compared condition estimates based on all sampled wetland types (EH, PRLH, PRLW) occurring in the XER and WMT regions (Fig. 7). Patterns of condition were quite different between the two regions, with 60 ± 15% of the area in the WMT estimated to be in good condition and 76 ± 15% of the area in the XER designated in poor condition. Thus, the scale at which condition results are viewed can provide very different perspectives. The estimated wetland area is greater in the XER compared to the WMT ecoregion (Fig. 7), so this may be driving overall results for the portion of the inference population representing inland wetlands across the West (Fig. 6); however, mesic/montane systems currently appear to be in better condition than xeric systems based on the 2011 VMMI (Fig. 7). Nevertheless, when viewing these results, it is important to consider that the percent of the estimated target population area represented by the sampled sites is greater in the XER region (52%) than the WMT region (24%) (Table 12).

Going forward, beginning with NWCA 2016, the survey design has been adjusted to increase the number of potential sample sites in the West (Olsen et al. 2019). A larger sample of sites could better represent wetland types across the West and enhance the precision of condition evaluation (i.e., reduce the margin of error surrounding the estimates) and will hopefully facilitate identification of a greater number of reference sites with limited levels of disturbance. Larger sample sizes should also allow development of specific condition thresholds for the NWCA aggregated wetland types (Table 1) for the Western Mountains and Valleys and for the Xeric West.

## Summary and next steps

### National-scale VMMI

We developed a VMMI applicable to diverse wetland types across the conterminous US (Table 1), and which provides a standardized approach to assessing wetland biological condition based on vegetation. The national VMMI includes four component metrics: a floristic quality index, relative importance of native species, richness of disturbance-tolerant species, and relative cover of native monocots (Table 7). This 4-metric VMMI was selected, from among numerous candidate VMMIs, based on the combined consideration of several performance criteria including sensitivity, repeatability, precision, minimization of metric redundancy, and metric identity (Table 6). The final VMMI was also strongly responsive, significantly distinguishing least from most disturbed sites for all 10 reporting groups (Table 11). Condition status was designated for each sampled site based on its VMMI value and the reporting group-specific condition thresholds associated with the site (Table 10).

In addition to utility as an indicator of biological condition for national and reporting group scales, the NWCA VMMI has potential for use at smaller scales within the conterminous US. The national VMMI is not intended to replace existing state, regional, or wetland type VMMIs that may reflect specific wetland attributes or be tailored to specific wetland types or state and regional management needs; however, for states or regions currently without a vegetation index for assessing biological condition, it may be a useful tool (or starting point) for supporting wetland monitoring activities. The NWCA VMMI can be calculated for other wetland sites in the conterminous US, using the defined metric scoring for the four component metrics (Table 8), the formula for VMMI calculation (sum of metric scores X 10/4), and the reporting group thresholds for condition categories (Table 10). This assumes availability of appropriate plant data and use of NWCA trait information or traits derived using similar procedures. Estimates of wetland area in particular condition categories require the use of a probability survey design (e.g., GRTS or other random sample) and sample-weights that reflect the wetland area represented by each site.

Finally, with the next iteration of the NWCA, the 2011 national VMMI will be reevaluated in light of (1) expanded sampling effort in some reporting groups and the addition of reporting groups for the West, (2) characteristics of additional reference sites, and (3) emerging new metrics. Results of this effort will determine whether modifications to the VMMI are needed to further enhance its utility. Initial components of this evaluation will likely involve repeating the VMMI development process described in this paper to determine: (1) whether the best performing national VMMI based on 2016 data will have the same or a different set of component metrics as the 2011 VMMI and (2) how thresholds for good, fair, and poor condition will be influenced by the addition of more reference sites and reporting groups.

### Baseline assessment of wetland condition

The 2011 NWCA resulted in the first-ever continental-scale assessment of wetland condition. Results provide a baseline description of wetland quality for the sampled wetland population across the conterminous US with 48 ± 5.1% (12.14 ± 1.42 million ha) of the evaluated resource estimated to be in good condition, 20 ± 4.49% (4.93 ± 1.25 million ha) in fair condition, and 32 ± 4.76% (8.10 ± 1.37 million ha) in poor condition. Condition results varied among the 10 reporting groups, providing information about levels of impact and potential vulnerabilities at regional and wetland type scales. The 2011 baseline for biological condition can help inform decision-making regarding use, management, and conservation of wetland resources and suggest future research priorities. Some additional insights to ecological implications of the 2011 NWCA results can be found in other work, including (1) characterization of key stressors to wetland condition (e.g., hydrological and physical disturbances (Lomnicky et al. 2019), nonnative plants (Magee et al. 2019), and soil heavy metals (Nahlik et al. 2019)) at national and NWCA reporting group scales and (2) evaluation of relationships between biological condition and specific stress indicators (Herlihy et al. 2019b; Herlihy et al. 2019c).

Future iterations of the NWCA, planned at 5-year intervals, will allow analysis of changes and trends in wetland condition. Comparison of the 2011 results with subsequent NWCA surveys will permit tracking of whether the biological condition of the wetland resource is improving (i.e., moving to good condition) or declining (i.e., moving to poor condition).

## Electronic supplementary material

ESM 1 (PDF 261 kb)
