# Supplementary material for: A national-scale vegetation multimetric index (VMMI) as an indicator of wetland condition across the conterminous United States
Source: Environ Monit Assess. 2019 Jun 20;191(Suppl 1):322. doi: 10.1007/s10661-019-7324-4 (PMC6586711; doi:10.1007/s10661-019-7324-4)
Supplement: Supplementary file 1 — (PDF 261 kb) [file 10661_2019_7324_MOESM1_ESM.pdf]

Online Resource 1 – Grayscale version of study area map

Paper title: A national-scale vegetation multimetric index (VMMI) as an indicator of wetland condition across the conterminous United States.

Journal: Environmental Monitoring and Assessment

Authors: Teresa K. Magee, Karen A. Blocksom, and M. Siobhan Fennessy

Corresponding author: Teresa K. Magee, U.S. Environmental Protection Agency, Office Research and Development, National Health Effects Laboratory, Western Ecology Division, Corvallis, Oregon

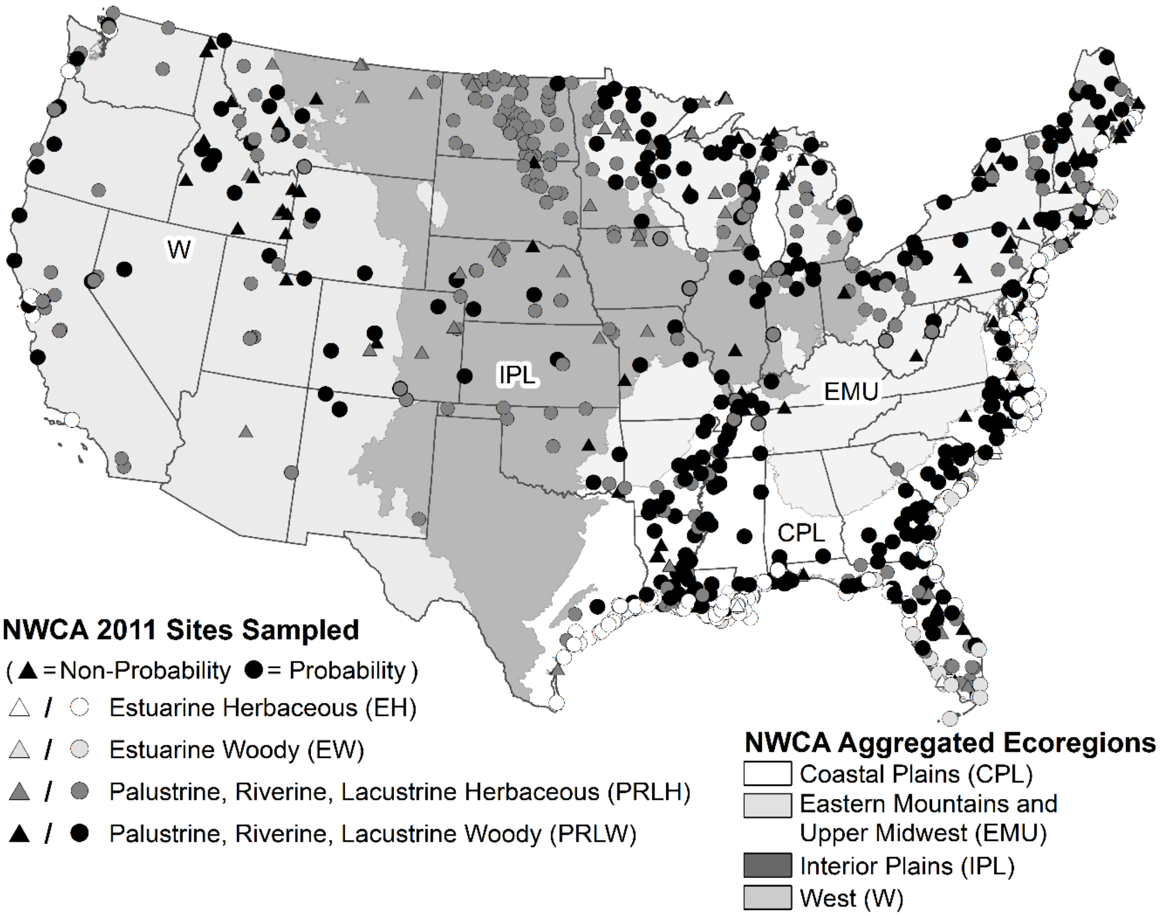

Online Resource 1 Grayscale version of study area map (Fig. 1 in paper). Locations of sites sampled in the 2011 National Wetland Condition Assessment (NWCA) by Aggregated Wetland Type within the four NWCA Ecoregions. Note, due to map scale and site proximity, individual sites are occassionally obscured by symbols for other sites.
